# Transcriptional Comparison of New Hybrid Progenies and Clone-Cultivars of Tea (*Camellia sinensis* L.) Associated to Catechins Content

**DOI:** 10.3390/plants11151972

**Published:** 2022-07-29

**Authors:** Hani Widhianata, Panjisakti Basunanda, Supriyadi Supriyadi, Taryono Taryono

**Affiliations:** 1 Faculty of Agriculture, Universitas Gadjah Mada, Jl. Flora, Caturtunggal, Depok, Sleman, Yogyakarta 55281, Indonesia; hani.widhianata86@gmail.com (H.W.); panjisakti@ugm.ac.id (P.B.); 2 Faculty of Agricultural Technology, Universitas Gadjah Mada, Jl. Flora, Caturtunggal, Depok, Sleman, Yogyakarta 55281, Indonesia; suprif248@ugm.ac.id; 3 Agrotechnology Innovation Center, Universitas Gadjah Mada, Jl. Tanjung Tirto, Kalitirto, Berbah, Sleman, Yogyakarta 55573, Indonesia

**Keywords:** catechin, heterosis, hybrid, tea, transcriptomic

## Abstract

Heterosis or hybrid vigor is the improved performance of a desirable quality in hybrid progeny. Hybridization between high-productive Assam type and high-quality Chinese type clone-cultivar is expected to develop elite tea plant progenies with high quality and productivity. Comparative transcriptomics analyses of leaves from the F1 hybrids and their parental clone-cultivars were conducted to explore molecular mechanisms related to catechin content using a high-throughput next-generation RNA-seq strategy and high-performance liquid chromatography (HPLC). The content of EGCG (epigallocatechin gallate) and C (catechin) was higher in ‘Kiara-8’ × ‘Sukoi’, ‘Tambi-2’ × ‘Suka Ati’, and ‘Tambi-2’ × ‘TRI-2025’ than the other hybrid and clone-cultivars. KEGG (Kyoto Encyclopedia of Genes and Genomes) and GO (Gene Ontology) analysis found that most pathways associated with catechins content were enriched. Significant differentially expressed genes (DEGs) mainly associated with phenylpropanoid, flavonoid, drug metabolism-cytochrome P450, and transcription factor (MYB, bHLH, LOB, and C2H2) pathways appeared to be responsible for the high accumulation of secondary metabolites in ‘Kiara-8’ × ‘Sukoi’, ‘Tambi-2’ × ‘Suka Ati’, and ‘Tambi-2’ × ‘TRI-2025’ as were detected in EGCG and catechin content. Several structural genes related to the above pathways have been obtained, which will be used as candidate genes in the screening of breeding materials.

## 1. Introduction

Tea (*Camellia sinensis* (L.) O. Kuntze) is a perennial plantation crop grown in more than 60 countries for the production of the most popular non-alcoholic beverage, tea. Global statistics indicate that approximately five million tons of tea leaves are harvested annually, and more than two billion cups are consumed daily [1]. Tea quality is a complex characteristic that exhibits tremendous genetic variability due to the accumulation of an array of secondary metabolites [2]. Due to awareness and multiple health benefits of tea, quality remains a key target for its genetic improvement [3,4].

Tea breeders in different tea-growing countries have developed anthocyanin-rich purple-colored tea varieties [3,5]. A recent study revealed that the antioxidant values of purple-leaf tea were higher than green-leaf tea due to the presence of catechins and anthocyanins [5,6]. Therefore, tea from anthocyanin-rich cultivars may become specialty tea with higher antioxidant activity [6]. Hybridization between clone-cultivars Assam type that has green-leaf and Chinese type that have purple-leaf are expected to develop new hybrid progenies with a combination of morphological characteristics that have high productivity and quality.

New tea cultivars have been developed through conventional breeding methods. The main breeding approaches in the tea breeding system are controlled hybridization and individual selection. Controlled hybridization and individual selection have been performed based on phenotypic evaluations. In breeding a clonal crop, such as tea, the selection is a two-stage process. In the first stage, the highest yielding individual seedlings are chosen; these are then propagated and planted in clone trials in the second stage [7]. Integration of breeding tools is a powerful strategy to enhance the efficiency of the breeding programs with an early screening of genotypes, which leads to the release of a new, improved cultivar within a comparatively short period of time unlike conventional tools [8].

Heterosis is widely used in plant breeding programs. Heterosis is the phenomenon in which a hybrid progeny has greater biomass and higher yield and quality than the parents; volatile heterosis was obvious in F1 hybrids [9]. The catechins and free amino acids in hybrids showed negative heterosis compared to its maternal cultivar TGY [10]. Earlier, we crossed Chinese-type clone-cultivars ‘Sukoi’, ‘Tambi-1’, and ‘Tambi-2’ with Assam-type clone-cultivars ‘TRI-2025’, ‘Suka Ati-40’, and ‘Kiara-8’ and produced some hybrid progenies. We selected five hybrid progenies that showed high potential yield for transcriptomic study related to catechins content and compared them to the parents, which were widely planted (Tambi-2 and TRI-2025). The Chinese-type parents exhibited unique morphological characteristics such as dark purple pigmentation with dense pubescence in shoot leaves. The Assam-type parents showed morphological characteristics such as broad leaves, lack of pigmentation in shoot leaves and petiole, with pubescence. Characterization of hybrid progeny is an initial step toward proper utilization of genetic resources [11]. We used morphological, biochemical, and molecular approaches to characterize hybrid progenies and compare them to the parent.

Cell–cell differences are determined by the expression of different sets of genes. All such proteins are often called transcription factors (TFs). Each TF has a specific DNA binding domain that recognizes a 6–10 base pair motif in the DNA [12]. Transcription factors regulate a complex network of gene expression. A better understanding of the transcription factors and their shared interactions among a set of co-regulated or differentially expressed genes can provide powerful insights into the key pathways governing such expression patterns.

The study of morphological characteristics [13], biochemical characteristics [14,15], molecular characteristics [16], and the diversity of tea is important for plant genetic improvement programs. Biochemical and DNA-based markers have been explored to improve quality traits in tea [17,18]. Although most of these studies have provided clear evidence that genotypic variation determines quality characteristics in tea, the molecular basis of these variations remains obscure. In this study, we investigated comparative transcriptomic and expressional regulation of putative functional genes in quality-related pathways between selected F1 hybrids and clone-cultivars. The results reported here provide a comprehensive analysis of transcriptomic differences that are reflected at the phenotypic level in tea plants. Because there have been limited reports comparing EGCG and C content among hybrid progenies and clone-cultivars as parents, these results are important for determining the relationship between genetic background and catechins content and the early study of catechins heterosis. Several structural genes have been obtained, which will be used as candidate genes in the screening of breeding materials

## 2. Results

### 2.1. Analysis of The Contents of Catechins

**Figure 1:**
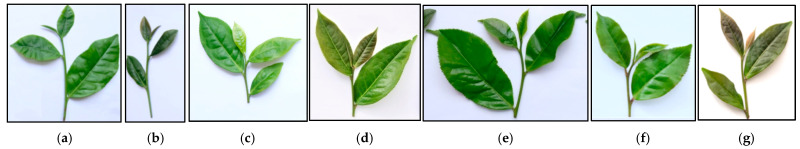
Shoot leaves of different tea clone-cultivars and progenies. (**a**) ‘TRI-2025’; (**b**) ‘Tambi-2’; (**c**) ‘Tambi-1’ × ‘Kiara-8’; (**d**) ‘Tambi-2’ × ‘Suka Ati-40’; (**e**) ‘Tambi-2’ × ‘Cinyiruan-143’; (**f**) ‘Tambi-2’ × ‘TRI-2025; (**g**) ‘Kiara-8’ × ‘Sukoi’.

The phenotypes of the ‘TRI-2025’ (green leaf), ‘Tambi-2’, ‘Tambi-1’ × ‘Kiara-8’, ‘Tambi-2’ × ‘Suka Ati-40’, ‘Tambi-2’ × ‘Cinyiruan-143’, ‘Tambi-2’ × ‘TRI-2025’, and ‘Kiara-8’ × ‘Sukoi’ were recorded (Figure 1). ‘TRI-2025’ was glossy, straight, with broad leaves and green. In contrast, the Tambi-2 exhibited unique morphological characteristics such as dark purple pigmentation in young shoots as well as young leaves and small leaves. Morphological traits such as leaf vestiture, anthocyanin pigmentation, immature leaf color, and petiole color have been determined in hybrids that were generated by the crossing of diverse parents. ‘Tambi-1’ × ‘Kiara-8’ and ‘Tambi-2’ × ‘Cinyiruan-143’ were glossy and green, ‘Tambi-2’ × ‘Suka Ati-40’ and ‘Tambi-2’ × ‘TRI-2025’ have had slightly anthocyanin pigmentation in immature leaf and have anthocyanin pigmentation in the petiole, while ‘Kiara-8’ × ‘Sukoi’ has more anthocyanin pigmentation in immature leaf and have anthocyanin pigmentation in the petiole.

**Figure 2:**
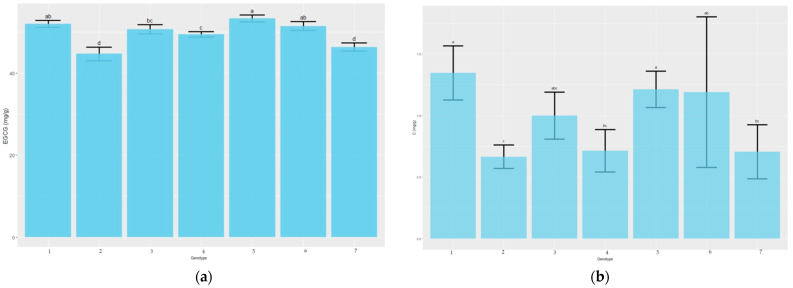
The content of EGCG and C. (**a**) EGCG content; (**b**). C content. 1: ‘Kiara-8’ × ‘Sukoi’, 2: ‘Tambi-1’ × ‘Kiara-8’, 3: ‘Tambi-2’, 4: ‘Tambi-2’ × ‘Cinyiruan-143’, 5: ‘Tambi-2’ × ‘Suka Ati-40’, 6: ‘Tambi-2’ × ‘TRI-2025’, 7: ‘TRI-2025’. Data are presented as mean ± SD of three independent biological replicates. Uppercase letter (a, b, c, and d) indicates significant difference (*p* < 0.05).

To explore the characteristic of catechin tea compounds in the ‘TRI-2025’, ‘Tambi-2’, and five hybrid progenies, we determined the contents of EGCG and C in these samples. HPLC analyses were conducted to determine the contents of EGCG and C in tea clone-cultivars and progenies (Figure 2). All standard compounds showed good linearity (R^2^ > 0.99) in a relatively wide concentration range. Moreover, distinct changes in the levels of flavor-related metabolites were detected in parents and the five hybrids. Analysis of individual catechins identified that EGCG is the most abundant catechin in all leaf samples. The content of EGCG was the highest for the ‘Tambi-2’ × ‘Suka Ati-40’ (53.302 mg/g), and it was not significantly different from ‘Kiara-8’ × ‘Sukoi’ (52.014 mg/g) and ‘Tambi-2’ × ‘TRI-2025’ (51.456 mg/g). ‘Tambi-2’ had lower EGCG content (50.703 mg/g), which was not significantly different from ‘Tambi-2’ × ‘Cinyiruan-143’ (49.466 mg/g), while ‘TRI-2025’ (46.365 mg/g) and ‘Tambi-1’ × ‘Kiara-8’ (44.667 mg/g) had the lowest EGCG content. The content of C in ‘Kiara-8’ × ‘Sukoi’ was the highest (1.34 mg/g), and it was not significantly different from ‘Tambi-2’ × ‘Suka Ati-40’ (1.21 mg/g), ‘Tambi-2’ × ‘TRI-2025’ (1.19 mg/g), and ‘Tambi-2’ (0.99 mg/g). ‘Tambi-1’ × ‘Kiara-8’ (0.67 mg/g) had the lowest C content (Figure 2).

In order to decode the genes involved in the differential phenotype in ‘TRI-2025’, ‘Tambi-2’, ‘Tambi-1’ × ‘Kiara-8’, ‘Tambi-2’ × ‘Suka Ati-40’, ‘Tambi-2’ × ‘Cinyiruan-143’, ‘Tambi-2’ × ‘TRI-2025’, and ‘Kiara-8’ × ‘Sukoi’, we de novo sequenced and assembled the transcriptome from leaf samples of the seven tea plants. The readings of clean reads of each sample remaining are presented in Supplementary Table S1. The GC content of the clean data was above 43%, and the quality score Q20 and Q30 percentage was above 97% and 93%, respectively (Supplementary Table S1).

The longest transcripts of each cluster were selected as unigenes. The total number of transcripts obtained was 333,310, and the total number of unigenes was 121,530, which, in turn, could be broken down into different groups of length intervals (Supplementary Table S2). The length distribution of transcripts and unigenes is shown in Supplementary Table S3. A total of 384,480,750 transcripts with a mean length of 1154 bp and an N50 length of 1649 bp were obtained. A total of 124,031,600 unigenes with a mean length of 1021 bp and an N50 length of 1477 bp were obtained.

### 2.2. Gene Functional Annotation and Classification

**Figure 3:**
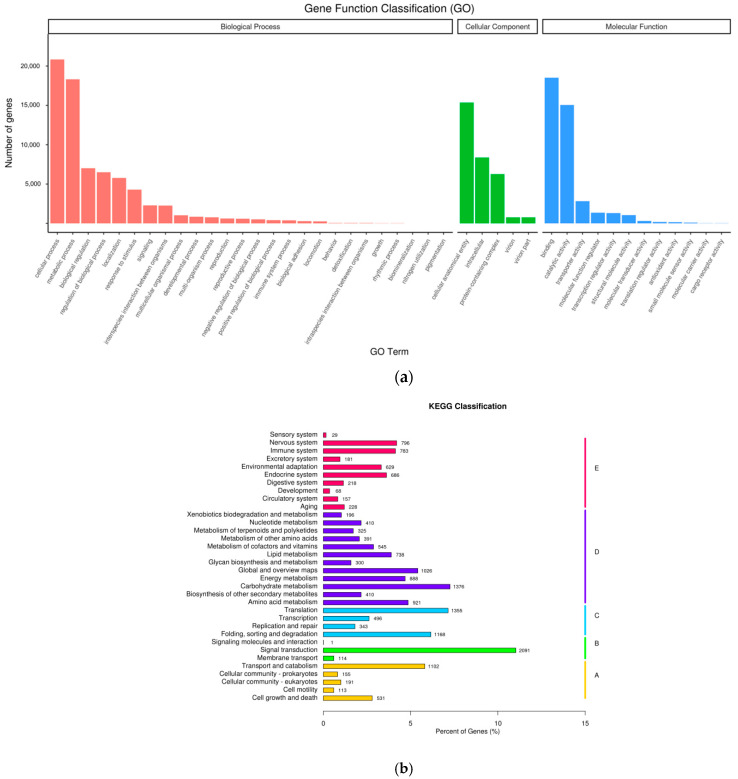
Gene Functional Annotation and Classification. (**a**) GO (Gene Ontology) classification; (**b**) KEGG (Kyoto Encyclopedia of Genes and Genomes) classification.

To achieve comprehensive gene functional annotation, seven databases were applied, resulting in a total of 121,530 functionally annotated genes (Supplementary Table S4). Gene functional classification is shown in Figure 3a. The cellular process and metabolic process were the most enriched GO terms, cellular anatomical entity, intracellular, and protein-containing complexes were the most enriched cellular component GO terms, while binding and catalytic activities were clearly enriched as molecular functions (Figure 3a). These results suggested that transcription factors (binding activity) and high enzymatic activity were involved in the biosynthesis of samples. The genes successfully annotated in KEGG could further be classified according to the KEGG pathway they were joined in. The metabolic pathways were mainly related to carbohydrate metabolism, amino acid metabolism, lipid metabolism, metabolism of cofactors and vitamins, biosynthesis of another secondary metabolism, nucleotide metabolism, and metabolism of terpenoids and polyketides (Figure 3b).

### 2.3. Transcription Factor Analysis

Transcription factors (TFs) are well known to play a crucial role in secondary metabolite biosynthesis in plants [19]. We searched for genes encoding TFs and obtained a total of 2348 TFs classified into various families. The five most transcription factor analysis results were GNAT, LOB, bHLH, C2H2, and MYB family (Supplementary Table S5).

### 2.4. Gene Expression Analysis

To understand gene expression between samples, we measured transcript levels in terms of fragments per kilo-base exon per million reads (FPKM) based on RNAseq data. Genes were called Differentially Expressed Genes (DEGs) if they exhibited at least a 2-fold change in transcript abundance using edgeR (*p* < 0.005). De novo transcriptome filtered by Corset was used as a reference [20].

### 2.5. Differential Expression Analysis

**Figure 4:**
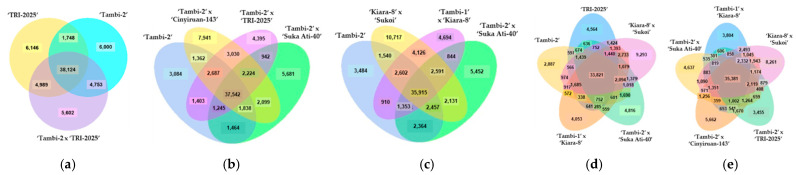
Venn diagram comparing differentially expressed genes between samples. Co-expression of (**a**) ‘TRI-2025’, ‘Tambi-2’, and ‘Tambi-2’ × ‘TRI-2025’, (**b**) ‘Tambi-2’, ‘Tambi-2’ × ‘Cinyiruan-143’, ‘Tambi-2’ × ‘TRI-2025’, and ‘Tambi-2’ × ‘Suka Ati-40’, (**c**) ‘Tambi-2’, ‘Kiara-8’ × ‘Sukoi’, ‘Tambi-1’ × ‘Kiara-8’, and ‘Tambi-2’ × ‘Suka Ati-40’, (**d**) ‘Tambi-2’, ‘TRI-2025’, ‘Kiara-8’ × ‘Sukoi’, ‘Tambi-2’ × ‘Suka Ati-40’, and ‘Tambi-1’ × ‘Kiara-8’, (**e**) ‘Tambi-2’ × ‘Suka Ati-40’, ‘Tambi-1’ × ‘Kiara-8’, ‘Kiara-8’ × ‘Sukoi’, ‘Tambi-2’ × ‘TRI-2025’, and ‘Tambi-2’ × ‘Cinyiruan-143’.

To uncover the genes involved in different samples, the gene expression values expressed as FPKM were compared between samples. The Venn diagram presents the number of genes that are uniquely expressed differentially within each group, with the overlapping regions showing the number of genes that are expressed in two or more groups (Figure 4). The Venn diagram depicts common and unique genes from a differentially expressed gene.

**Figure 5:**
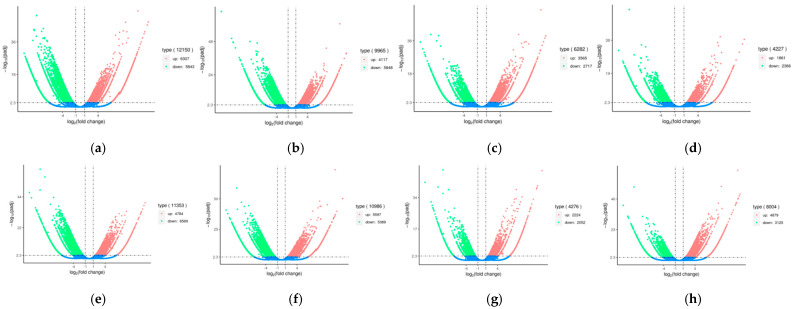

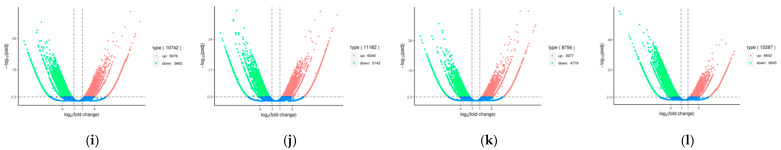
Volcano plots of differentially expressed genes between pairwise comparison samples. (**a**) ‘TRI-2025’ vs. ‘Tambi-2’; (**b**) ‘Tambi-2’ × ‘Suka Ati’ vs. ‘Tambi-2’; (**c**) ‘Tambi-2’ × ‘Cinyiruan-143’ vs. ‘Tambi-2’; (**d**) ‘Tambi-2’ × ‘TRI-2025’ vs. ‘Tambi-2’; (**e**) ‘Kiara-8’ × ‘Sukoi’ vs. ‘Tambi-2’; (**f**) ‘Tambi-1’ × ‘Kiara-8’ vs. ‘Tambi-2’; (**g**) ‘Tambi-2’ × ‘TRI-2025’ vs. ‘Tambi-2’ × ‘Suka Ati-40’; (**h**) ‘Tambi-2’ × ‘Cinyiruan-143’ vs. ‘Tambi-2’ × ‘Suka Ati-40’; (**i**) ‘Tambi-2’ × ‘TRI-2025’ vs. ‘Tambi-2’ × ‘Cinyiruan-143’; (**j**) ‘Tambi-2’ × ‘Suka Ati-40’ vs. ‘Kiara-8’ × ‘Sukoi’; (**k**) ‘Kiara-8’ × ‘Sukoi’ vs. ‘Tambi-2’ × ‘TRI-2025’; (**l**) ‘Kiara-8’ × ‘Sukoi’ vs. ‘Tambi-2’ × ‘Cinyiruan-143’.

Furthermore, volcano plots were generated to display the significant differences and up and down-regulated genes between pairwise comparison samples. DEGs of ‘Tambi-2’ were up-regulated compared to ‘TRI-2025’, ‘Tambi-2’ × ‘Cinyiruan-143’, and ‘Tambi-1’ × ‘Kiara-8’, while down-regulated compared to ‘Tambi-2’ × ‘Suka Ati-40’, ‘Tambi-2’ × ‘TRI-2025’, and ‘Kiara-8’ × ‘Sukoi’. DEGs of ‘Tambi-2’ × ‘Suka Ati-40’ were up-regulated compared to ‘Tambi-2’ × ‘TRI-2025’ and ‘Tambi-2’× ‘Cinyiruan-143’, while down-regulated compared to ‘Kiara-8’ × ‘Sukoi’. DEGs of ‘Tambi-2’ × ‘TRI-2025’ were up-regulated compared to ‘Tambi-2’ × ‘Cinyiruan-143’, while down-regulated compared to ‘Kiara-8’ × ‘Sukoi’ and ‘Tambi-2’ × ‘Suka Ati-40’. DEGs of ‘Kiara-8’ × ‘Sukoi’ were up-regulated compared to ‘Tambi-2’ × ‘Cinyiruan-143’, ‘Tambi-2’ × ‘TRI-2025’, and ‘Tambi-2’ × ‘Suka Ati-40’ (Figure 5).

### 2.6. KEGG Enrichment Analysis

**Figure 6:**
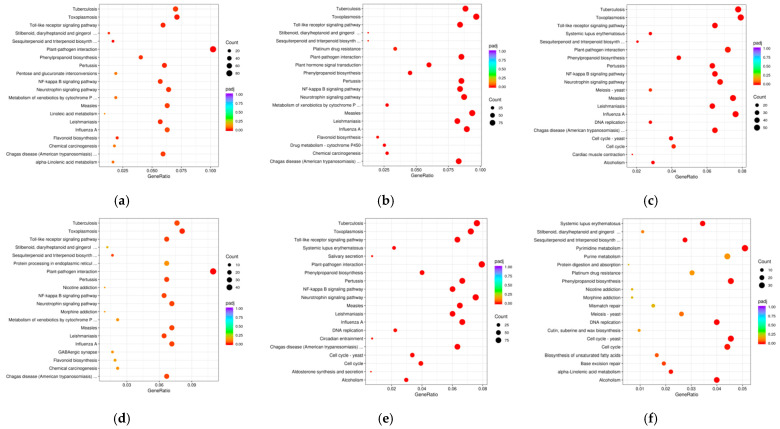

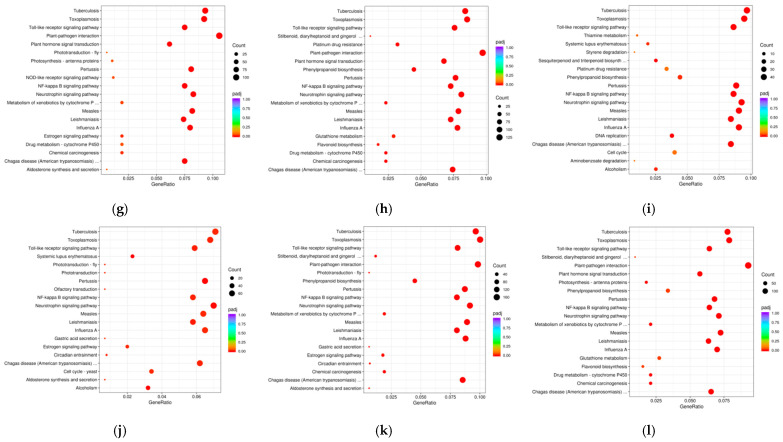
KEGG enrichment scattered plots analysis between pairwise comparison samples. (**a**) ‘TRI-2025’ vs. ‘Tambi-2’; (**b**) ‘Tambi-2’ × ‘Suka Ati-40’ vs. ‘Tambi-2’; (**c**) ‘Tambi-2’ × ‘Cinyiruan-143’ vs. ‘Tambi-2’; (**d**) ‘Tambi-2’ × ‘TRI-2025’ vs. ‘Tambi-2’; (**e**) ‘Kiara-8’ × ‘Sukoi’ vs. ‘Tambi-2’; (**f**) ‘Tambi-1’ × ‘Kiara-8’ vs. ‘Tambi-2’; (**g**) ‘Tambi-2’ × ‘TRI-2025’ vs. ‘Tambi-2’ × ‘Suka Ati-40’; (**h**) ‘Tambi-2’ × ‘Cinyiruan-143’ vs. ‘Tambi-2’ × ‘Suka Ati-40’; (**i**) ‘Tambi-2’ × ‘TRI-2025’ vs. ‘Tambi-2’ × ‘Cinyiruan-143’; (**j**) ‘Tambi-2’ × ‘Suka Ati-40’ vs. ‘Kiara-8’ × ‘Sukoi’; (**k**) ‘Kiara-8’ × ‘Sukoi’ vs. ‘Tambi-2’ × ‘TRI-2025’; (**l**) ‘Kiara-8’ × ‘Sukoi’ vs. ‘Tambi-2’ × ‘Cinyiruan-143’.

To identify the molecular mechanism underlying specific and secondary metabolites of tea, we carried out a KEGG pathway enrichment analysis using KOBAS v3.0. KEGG enrichment analysis of DEGs indicates that the translation and secondary metabolic processes dominated the difference in biological processes between pairwise comparison samples. The top 20 DEGs enriched pathways of each sample were displayed (Figure 6). The KEGG enrichment pathway shows that the DEGs significantly enriched the pathways. We have looked for gene clusters that were significantly different between pairwise comparison samples associated with tea quality.

**Figure 7:**
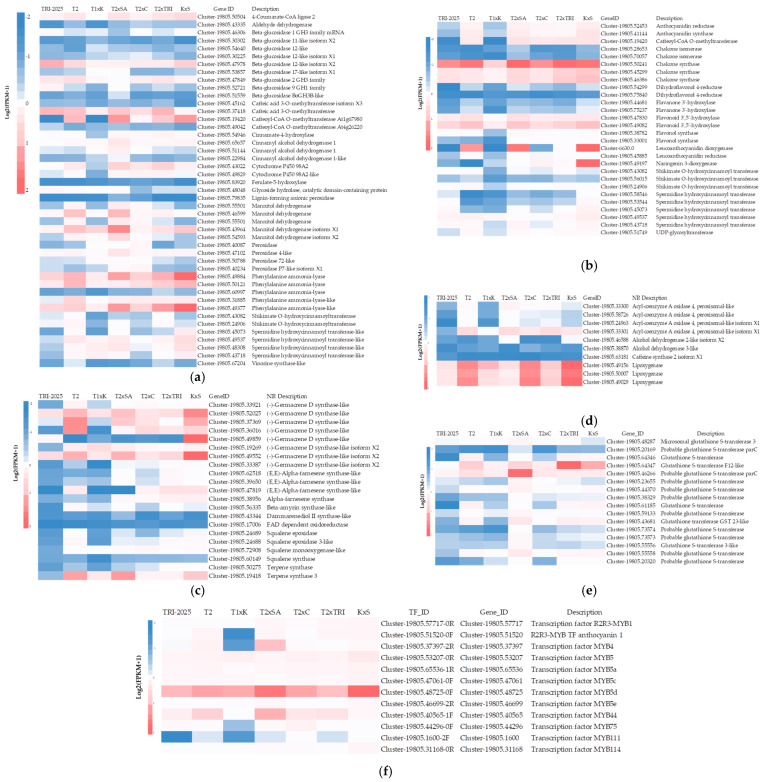
DEG enrichment analysis of each gene involved in biosynthesis and metabolism pathway that was associated with tea quality. (**a**) Phenylpropanoid biosynthesis; (**b**) flavonoid biosynthesis; (**c**) sesquiterpenoid and triterpenoid biosynthesis; (**d**) alpha-linolenic acid biosynthesis; (**e**) drug metabolism-cytochrome P450; (**f**) MYB TF.

In the present work, KEGG enrichment analysis of DEGs in the pairwise comparison of each sample showed that biosynthesis and metabolism pathways were enriched, and the significantly different pathways associated with tea quality were phenylpropanoid, flavonoid, sesquiterpenoid and triterpenoid biosynthesis, drug metabolism-cytochrome P450, and alpha-linolenic acid metabolism (Figure 6). Unigenes involved in these pathways were identified with FPKM values > 50 (Figure 7). We used KEGG mapping to map molecular objects to molecular interaction (Supplementary Figures S1–S5).

Flavonoids are a large and diverse group of secondary metabolites that are synthesized through a specific branch of a phenylpropanoid pathway [21]. Highly expressed structural genes involved in the phenylpropanoid biosynthesis identified were phenylalanine ammonia-lyase (PAL), 4-coumarate-CoA ligase 2 (4CL), beta-glucosidase-12 like isoform X2, caffeoyl-CoA O-methyltransferase At1g67980 (CCoAOMT), cinnamyl alcohol dehydrogenase 1 (CAD1), cytochrome P450 98A2, mannitol dehydrogenase, caffeic acid 3-O-methyltransferase (COMT), cinnamate-4-hydroxylase (C4H), and spermidine hydroxycinnamoyl transferase (SHT), which have shown differences in the number of DEGs among the analyzed samples (Figure 7a). Most of the genes encoding phenylpropanoid biosynthesis enzymes were identified and found to be differentially expressed in the shoot leaves of different tea clone-cultivars and hybrid progenies.

**Figure 8:**
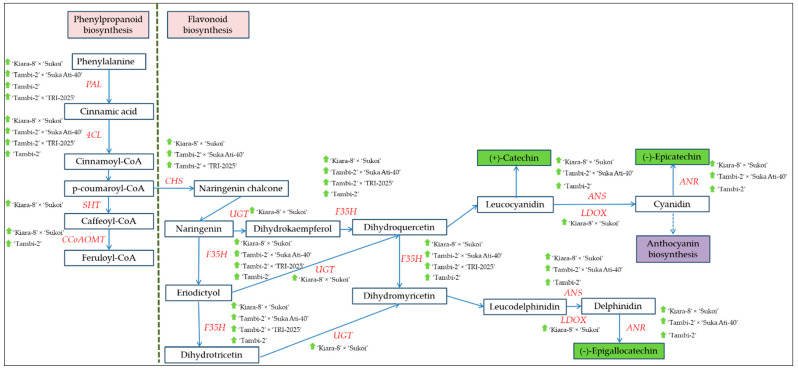
Diagram of the genes (red) influencing the formation of EGCG and C and their up-regulation (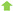 ) in hybrid progenies and clone-cultivars.

From DEGs enrichment analysis and KEGG mapping, PAL (Cluster-19805.49884, Cluster-1980549377, and Cluster-19805.50121) showed up-regulation in ‘Kiara-8’ × ‘Sukoi’, ‘Tambi-2’ × ‘Suka Ati-40’, ‘Tambi-2’, and ‘Tambi-2’ × ‘TRI-2025’ in the formation of cinnamic acid from phenylalanine. 4CL (Cluster-19805.50504) showed up-regulation in ‘Kiara-8’ × ‘Sukoi’, ‘Tambi-2’ × ‘Suka Ati-40’, ‘Tambi-2’ × ‘TRI-2025’, and ‘Tambi-2’ in the formation of cinnamoyl-CoA from cinnamic acid. SHT (Cluster-19805.45073, Cluster-19805.49537, Cluster-19805.48308, and Cluster-19805.43718) showed up-regulation in ‘Kiara-8’ × ‘Sukoi’ in the formation of caffeoyl-CoA from p-coumaroyl-CoA. CCoAOMT (Cluster-19805.19420) showed up-regulation in ‘Tambi-2’ × ‘Suka Ati-40’, ‘Kiara-8’ × ‘Sukoi’, and ‘Tambi-2’ in the formation of feruloyl-CoA from caffeoyl-CoA. Mannitol dehydrogenase isoformX1 (Cluster-19805.43964) showed up-regulation in ‘Tambi-2’ × ‘Suka Ati-40’ in the formation of p-coumaryl alcohol from p-coumaraldehyde (Figure 7a and Supplementary Figure S1). Figure 8 shows the genes that influence the formation of EGCG and C.

Highly expressed structural genes involved in flavonoid biosynthesis were identified in analyzed samples, chalcone synthase (CHS), flavonoid 3′,5′-hydroxylase (F35H), anthocyanidin synthase (ANS), anthocyanidin reductase (ANR), caffeoyl-CoA O-methyltransferase (CCoAOMT), leucoanthocyanidin dioxygenase (LDOX), spermidine hydroxycinnamoyl transferase (SHT), cinnamate-4-hydroxylase (C4H), UDP glycosyltransferase (UGT), flavonol synthase (FLS), and leucoanthocyanidin reductase (LAR) (Figure 7b). PAL, 4CL, CHS, CHI/CFI, F′3H, F3′5′H, ANS, and LAR genes are involved in the flavonoid pathway and showed a significant positive correlation with catechin content in tea; those genes with UGT may be associated with the modification and stabilization of these metabolites hence their abundance for enhancing flavor-related attributes in tea cultivars [2]. LAR, ANS, and ANR are downstream enzymes in the flavonoid biosynthetic pathway, which play key roles in determining individual catechins [22].

CHS (Cluster-19805.50241) showed up-regulation in ‘Tambi-2’ × ‘Suka Ati-40’, ‘Tambi-2’ × ‘TRI-2025’, ‘Tambi-2’, and ‘Kiara-8’ × ‘Sukoi’, and the other CHS (Cluster-19805.45299 and Cluster-19805.46386) showed up-regulation in ‘Kiara-8’ × ‘Sukoi’, ‘Tambi-2’ × ‘TRI-2025’, and ‘Tambi-2’ × ‘Suka Ati-40’ in the formation of naringenin chalcone from p-coumaroyl-CoA. F35H (Cluster-19805.49082) showed up-regulation in ‘Kiara-8’ × ‘Sukoi’, ‘Tambi-2’ × ‘Suka Ati-40’, ‘Tambi-2’, and ‘Tambi-2’ × ‘TRI-2025’ in the formation of eriodictyol and dihydroquercetin from naringenin and dihydrokaempferol, respectively, and dihydrotricetin and dihydromyricetin from eriodictyol and dihydroquercetin, respectively. ANS (Cluster-19805.41144) showed up-regulation in ‘Kiara-8’ × ‘Sukoi’, ‘Tambi-2’ × ‘Suka Ati-40’, and ‘Tambi-2’ in the formation of cyanidin and delphinidin from leucocyanidin and leucodelphinidin, respectively. ANR (Cluster-19805.52453) showed up-regulation in ‘Kiara-8’ × ‘Sukoi’, ‘Tambi-2’ × ‘Suka Ati-40’, and ‘Tambi-2’ in the formation of (-)-epigallocatechin and (-)-epicatechin from delphinidin and cyanidin, respectively. LDOX (Cluster-6630.0) showed up-regulation in ‘Kiara-8’ × ‘Sukoi’ in the formation of cyanidin and delphinidin from leucocyanidin and leucodelphinidin, respectively (Anthocyanin biosynthesis). SHT (Cluster-19805.49537) showed up-regulation in ‘Kiara-8’ × ‘Sukoi’ in the formation of caffeoyl-CoA from p-coumaroyl-CoA. UGT (Cluster-19805.51749) and naringenin-3-dioxygenase (Cluster-19805.49197) showed up-regulation in ‘Kiara-8’ × ‘Sukoi’ in the formation of dihydrokaempferol, dihydroquercetin, and dihydromyricetin from naringenin, eriodictyol, and dihydrotricetin, respectively (Figure 7b and Supplementary Figure S2).

Highly expressed structural genes involved in sesquiterpenoid biosynthesis were identified in analyzed samples, (-)-germacrene D synthase-like, alpha-farnesene synthase (AFS), squalene epoxidase 3 (SQE3), squalene monooxygenase (SM), and terpene synthase 3 (TPS3). (-)-Germacrene D synthase-like (Cluster-19805.49859 and Cluster-19805.52025) were up-regulated in ‘Kiara-8’ × ‘Sukoi’, while (-)-germacrene D synthase-like (Cluster-19805.37369 and Cluster-19805.36016) were up-regulated in ‘Tambi-2’ in the formation of (-)-germacrene D. AFS (Cluster-19805.47819 and Cluster-19805.38956) were up-regulated at ‘Tambi-2’ × ‘TRI-2025’ and ‘Kiara-8’ × ‘Sukoi’ in the formation of (E,E)-α-farnesene. TPS3 (Cluster-19805.19418) was up-regulated in ‘Tambi-2’ × ‘Suka Ati-40’, ‘Tambi-2’, and ‘Kiara-8’ × ‘Sukoi’ in the formation of (-)-germacrene D. (-)-Germacrene D synthase-like isoformX2 (Cluster-19805.49552) (Figure 7c and Supplementary Figure S3). In plants, a family of TPSs is responsible for the synthesis of the various terpene molecules from two isomeric 5-carbon precursor ‘building blocks’, leading 5-carbon isoprene, 10-carbon monoterpenes, 15-carbon sesquiterpenes, and 20-carbon diterpenes [23]. Methyl jasmonate resulted in the enhanced expression of the majority of *CsTPS* (terpenoid synthase gene) [24]. Methyl jasmonate (MeJA), a volatile organic compound, is a principal flowery aromatic compound in tea [25].

In the alpha-linolenic acid biosynthesis, acyl coenzyme A oxidase 4 (ACX) and lipoxygenase (LOX) showed a high expression level in analyzed samples. The number of DEGs of ACX (Cluster-19805.33301) was up-regulated in ‘Tambi-2’ × ‘Suka Ati-40’, ‘Tambi-2’ × ‘TRI-2025’, ‘Tambi-2’, ‘Kiara-8’ × ‘Sukoi’, and ‘Tambi-2’ × ‘Cinyiruan-143’ in the formation of trans-2-Enoyl-OPC6-CoA from OPC6-CoA. LOX (Cluster-19805.49156, Cluster-19805.50007, and Cluster-19805.49029) has a high expression in ‘Kiara-8’ × ‘Sukoi’, ‘Tambi-2’ × ‘Cinyiruan-143’, and ‘Tambi-2’ followed by ‘Tambi-2’ × ‘TRI-2025’, ‘Tambi-1’ × ‘Kiara-8’, and ‘TRI-2025’ in the formation of 9 (S)-HpOTrE and 13 (S)-HpOTrE from alpha-linolenic acid (Figure 7d and Supplementary Figure S4). Oxylipins, including jasmonic acid (JA) and volatiles, are important for signaling in plants, and these are formed by the lipoxygenase (LOX) enzyme family [26]. β-glucosidases, ADH, and LOX are associated with the biosynthesis of aroma and flavor-forming compounds [2].

DEG enrichment analysis in drug metabolism-cytochrome P450 showed that glutathione S-transferase genes dominated and showed high expression between samples. Cluster-19805.64347 (glutathione S-transferase F12-like) showed a high expression level in ‘Tambi-2’ × ‘TRI-2025’, ‘Kiara-8’ × ‘Sukoi’, ‘Tambi-2’ × ‘Suka Ati-40’, and ‘Tambi-2’, while Cluster-19805. 46266 (probable glutathione S-transferase) showed the highest expression level in ‘Tambi-2’ × ‘Suka Ati-40’ (Figure 7e) in the formation of 4-Glutathionyl-CPA from Aldophosphamide (Supplementary Figure S5). Glutathione S-transferases (CsGSTs) of *C. sinensis* were positively correlated with the accumulation of flavonols, anthocyanins, and proanthocyanins in tea tissues and cultivars [27].

Annotation analysis in the analyzed sample and DEG analysis showed a high DEG number of MYB transcription factor families, Cluster-19805.57717 (R2R3-MYB1), Cluster-19805.51520 (R2R3 –MYB transcription factor anthocyanin1), Cluster-19805.37397 (MYB4), Cluster-19805.53207 (MYB5), Cluster-19805.65536 (MYB5a), Cluster-19805.47061 (MYB5c), Cluster-19805.48725 (MYB5d), Cluster-19805.46699 (MYB5e) Cluster-19805.40565 (MYB44), Cluster-19805.44296 (MYB75), Cluster-19805.1600 (MYB111), and Cluster-19805.31168 (MYB114) (Figure 7f). Seven TFs, including those mentioned above, were identified as having possible crucial roles in controlling the transcriptomic regulation of flavonoids [28].

## 3. Discussion

The flavonoid biosynthesis pathway is highly organized. Key structural genes and numerous transcription factors act as regulatory protein modulators of gene expression through sequence-specific DNA binding at the transcriptional level or by protein–protein interactions during chromatin remodeling [29]. In our transcriptomic data, DEGs of some genes and transcription factors related to phenylpropanoid, flavonoid, and sesquiterpenoid biosynthesis showed different expression levels in ‘TRI-2025’, ‘Tambi-2’, ‘Tambi-1’ × ‘Kiara-8’, ‘Tambi-2’ × ‘Suka Ati-40’, ‘Tambi-2’ × ‘Cinyiruan-143’, ‘Tambi-2’ × ‘TRI-2025’, and ‘Kiara-8’ × ‘Sukoi’.

Enrichment analysis revealed that 10 genes were involved in the catechin biosynthesis of *C. sinensis* var. *sinensis* [30]. However, we have detected ANR, ANS, CCoAOMT, CHI, CHS, C4H, Cytochrome P450 98A2, DFR, F3H, F35H, FLS, LAR, LDOX, Shikimate O-hydroxycinnamoyl transferase, SHT, and UGT in the flavonoid biosynthesis. The principal DEGs of genes CHS, F35H, and SHT presented high expression levels in ‘Kiara-8’ × ‘Sukoi’, ‘Tambi-2’ × ‘Suka Ati-40’, and ‘Tambi-2’ × ‘TRI-2025’, ‘Tambi-2’, and also ‘TRI-2025’ (Figure 7b). CHS is a key enzyme that catalyzes the first committed step in the flavonoid biosynthetic pathway [31]. F35H is a key controller of tri-hydroxyl flavan-3-ol synthesis in tea plants, which can effectively convert 4′-hydroxylated flavanones into 3′4′5′- and/or 3′4′-hydroxylated products [11].

Phenylalanine is the initial substrate of the phenylpropanoid pathway, which is determined by the catalysis PAL, the first and rate-limiting enzyme that regulates overall carbon flux into phenylpropanoid metabolism due to its unique metabolic position [32]. Flavonoids are synthesized via the phenylpropanoid pathway through the transformation of phenylalanine into 4-Coumarate-CoA, which ultimately enters the flavonoid biosynthesis pathway [33]. 4-Coumarate-CoA is one of the main precursors for flavonoids that produce chalcones by CHS [34]. 4-Coumarate-CoA is synthesized through the catalytic activity of 4CL [35]. In our sample analysis, DEGs of PAL (Cluster-19805.49884, Cluster-19805.49377, and Cluster-19805.50121) in ‘Kiara-8’ × ‘Sukoi’ and ‘Tambi-2’ × ‘Suka Ati-40’ were higher compared to that in Tambi-2 as a purple-leaf cultivar. DEGs of 4CL in ‘Kiara-8’ × ‘Sukoi’, ‘Tambi-2’ × ‘Suka Ati-40’, and ‘Tambi-2’ × ‘TRI-2025’ were higher compared to that Tambi-2 (Figure 7b). The DEG analysis was related to the content of EGCG and catechin in the samples, EGCG and catechin content of ‘Tambi-2’ × ‘Suka Ati-40’, ‘Kiara-8’ × ‘Sukoi’, and ‘Tambi-2’ × ‘TRI-2025’ were higher compared to that in ‘Tambi-2’ (Figure 2).

DEGs of C4H in ‘Kiara-8’ × ‘Sukoi’, ‘Tambi-2’ × ‘Cinyiruan-143’, ‘Tambi-2’ × ‘Suka Ati-40’, and ‘Tambi-2’ × ‘TRI-2025’ were higher compared to ‘TRI-2025’ and ‘Tambi-2’ (Figure 7a). PAL, C4H, and 4CL are the three basic enzymes of phenylpropanoid metabolism in which 4CL is an important enzyme of the pathway that catalyzes the formation of 4-coumaroyl-CoA in the presence of ATP and Mg^2+^, 4-coumaroyl-CoA serves as a precursor for the biosynthesis of numerous plant secondary products, such as flavonoids [36]. DEGs of Cytochrome P450 98A2 in ‘Tambi-2’ × ‘Suka Ati-40’, ‘Tambi-2’ × ‘TRI-2025’, and ‘Kiara-8’ × ‘Sukoi’ were higher compared to ‘Tambi-2’ (Figure 6). Cytochrome P450 and C4H catalyzed the central reaction of three catalytic steps leading from phenylalanine to activated 4-coumaroyl CoA controls the flux of metabolites toward all branches of the pathway [37].

The DEGs of Caffeoyl CoA O-methyltransferase had the highest expression level in ‘Tambi-2’ × ‘Suka Ati-40’, ‘Kiara-8’ × ‘Sukoi’, ‘Tambi-2’, and ‘Tambi-2’ × ‘TRI-2025’ (Figure 7a). The overexpression of Caffeoyl CoA O-methyltransferase indicates increased phenolic deposition within cell walls [38]. The highest number of caffeic acid 3-O-methyltransferase (COMT) DEGs was detected in ‘Tambi-2’ × ‘TRI-2025’ followed by ‘TRI-2025’, ‘Tambi-2’, ‘Tambi-2’ × ‘Suka Ati-40’, and ‘Tambi-2’ × ‘Cinyiruan-143’ (Figure 7a). COMT and CCoAOMHT had roles in the biosynthesis of lignin, flavonoid, and sinapoyl malate [39]. Mannitol dehydrogenase in ‘Tambi-2’ × ‘Suka Ati-40’ was the highest compared to other clone-cultivars and progenies (Figure 7a). Mannitol dehydrogenase participates in the regulation of nutrients or energy metabolism and fatty acid metabolism by affecting ammonium phenylpropionate metabolism, endoplasmic reticulum protein processing, and starch and sugar metabolism pathways [40].

ANR, ANS, CHS, CHI, DFR, F3H, F35H, LAR, and UGT detected in the samples were some key enzymes that directly regulated the flavonoid biosynthesis in tea. The DEGs of those genes had higher expression numbers at ‘Kiara-8’ × ‘Sukoi’, ‘Tambi-2’ × ‘Suka Ati-40’, and ‘Tambi-2’ than the other samples. ANR is one of the two enzymes of the flavonoid biosynthesis pathway that produces flavan-3-ols (epicatechin) monomers from anthocyanidin [41]. ANS is the last key enzyme in anthocyanin synthesis, directly affecting anthocyanin accumulation [42]. Interestingly, the gene upstream and downstream in the flavonoid biosynthesis pathway differed significantly among the different tea clone-cultivars and progenies. The downstream enzymes LAR and ANR are responsible for the conversion of anthocyanidin to catechin and epicatechin [43,44]. UDP-Glycosyltransferase (UGT), the final enzyme in anthocyanin biosynthesis, catalyzes glucosyl transfer from UDP-glucose to 3-hydroxyl group to form stable cyanine glucosides [42]. The expression of CsLAR in tobacco overproducing anthocyanin led to the accumulation of higher levels of epicatechin and its glucoside than of catechin [45].

The purple leaves of ‘Kiara-8’ × ‘Sukoi’, ‘Tambi-2’ × ‘Suka Ati-40’, and ‘Tambi-2’ × ‘TRI-2025’ showed a high content of anthocyanin. The increase in anthocyanin biosynthesis was mainly due to increased PAL activity [42]. Cluster-19805.41144 (ANS) was up-regulated in ‘Kiara-8’ × ‘Sukoi’ and ‘Tambi-2’ × ‘Suka Ati-40’ progenies than those in ‘Tambi-2’ cultivar, wherein this gene has played a role in the formation of Cyanidin and Delphinidin from Leucocyanidin and Leucodelphinidin in anthocyanin biosynthesis. The abundances of anthocyanin synthesis-related enzymes, such as CHS, CHI, DHR, and ANS, as well as anthocyanin accumulation-related UDP-glucosyl transferase and ATP-binding cassette (ABC) transporters in the purple leaves, were all significantly higher than those in the green leaves [36].

R2R3-MYB families (Cluster-19805.57717 and Cluster-19805.51520) were identified in the samples that had high DEG numbers in ‘Kiara-8’ × ‘Sukoi’, ‘Tambi-2’, and ‘Tambi-2’ × ‘Suka Ati-40’ (Figure 7f). An R2R3-MYB transcription factor (CsMYB6A) can activate the expression of flavonoid-related structural genes, especially CHS and 3GT, controlling the accumulation of anthocyanins in the leaf [46]. In this study, the majority of identified DEGs encoding TFs beside MYB families were highly expressed, including bHLH, LOB, and C2H2 (Supplementary Table S5). A complex of MYB-bHLH-WDR (MBW) shows action in order to trigger the structural genes responsible for the process of flavonoid biosynthesis [47]. The C2H2-zinc finger protein (C2H2-ZFP) is essential for the regulation of plant development and is widely responsive to diverse stresses, including drought, cold, salt stress, and flavonoid accumulation, in higher plants [48]. The lateral organ boundary (LOB) domain (LBD) genes play important roles in abiotic stress and flavonoid biosynthesis [49].

This study showed the catechin heterosis, especially in EGCG content in ‘Tambi-2’ × ‘TRI-2025’ compared with the female (‘Tambi-2’) and male parents (‘TRI-2025’) and ‘Tambi-2’ × ‘Suka Ati-40’ compared with the female parent (‘Tambi-2’). Several gene clusters and TFs that influence the formation of EGCG and C in the biosynthesis of phenylpropanoids and flavonoids have been identified.

Transcriptomic variation and expressional regulation of putative functional genes in quality-related pathways between green-leaf and purple-leaf clone-cultivars and selected F1 hybrids from crossing between clone-cultivar parents were detected using a comprehensive analysis of transcriptomic differences that are reflected at the phenotypic level in tea plants. ‘Kiara-8’ × ‘Sukoi’, ‘Tambi-2’ × ‘Suka Ati’, and ‘Tambi-2’ × ‘TRI-2025’ were selected as candidate high-quality tea hybrids based on EGCG and catechin content and DEGs involved in phenylpropanoid, flavonoid, and drug metabolism-cytochrome P450 pathways, and also high expression level of TF (MYB, bHLH, LOB, and C2H2) related to the phenylpropanoid and flavonoid pathways. These results are important for determining the relationship between genetic background and the catechin content of tea to improve future breeding materials.

## 4. Materials and Methods

### 4.1. Plant Materials and Sample Collection

For transcriptomic and biochemical analysis, we used an individual plant of parent cultivars ‘Tambi-2’ (Chinese type) and ‘TRI-2025’ (Assam type) and also a series of F1-hybrids: ‘Tambi-2’ × ‘TRI-2025’, ‘Tambi-2’ × ‘Suka Ati’, ‘Tambi-2’ × ‘Cinyiruan-143’, ‘Tambi-1’ × ‘Kiara-8’, and ‘Kiara-8’ × ‘Sukoi’ from crossing between parents with green-leaf tea (‘TRI-2025’, ‘Kiara-8’, and ‘Suka Ati-40’) and purple-leaf tea (‘Tambi-1’, ‘Tambi-2’, and ‘Sukoi’) were collected in September 2020 from a polyclonal garden of PT Pagilaran in Batang district, Central Java province. Fresh and healthy leaves were harvested and immediately frozen in liquid nitrogen after collection. These preparations were preserved at −80 °C in a container in the laboratory before being processed further for secondary metabolites and RNA extraction.

### 4.2. Extraction and Quantification of Catechins

To determine major constituents in tea, fresh leaves were crushed into a fine powder with liquid nitrogen using a mortar and pestle. About 1 g of fine powder was dissolved in 10 mL methanol. Further, the solution was subjected to a continuous vortex for 24 h and subsequently centrifuged at 2000 rpm (15 min). The supernatant was then filtered through 5C filter paper. The solution was then collected in small bottles and evaporated to receive the crude extract. About 1.5 mg of crude extract was dissolved in 1.5 mL methanol for HPLC. The solution was subjected to a continuous vortex for 5 min. The solution was then filtered through a 0.45-micron syringe filter (nylon) into an HPLC vial screw. The standard solution of EGCG and catechin (C) was diluted in the mobile phase, yielding the standard solutions with different concentrations. Subsequently, the solution was filtered using a 0.45-micron syringe filter into an HPLC vial screw. Sample-extracted solutions and standard solutions were later degassed by sonication for 15 min before injecting into the HPLC system to obtain the chromatograms. The standard and sample-extracted solutions (1 mg/mL) were run to ensure that both analytes were chemically identical with similar molecular structures. This analysis was performed on a high-pressure liquid chromatograph (Shimadzu Prominence Detector SPD-M20A) installed with Analyst software. A C18 column (Shim-pack GIST 150 mm × 4.6 mm, 5 µm) was used at a flow rate of 1.0 mL/min. The detection wavelength was set to 200–600 nm, and the column temperature was 25 °C. The mobile phase consists of MeOH: 0.05% TFA aq 25:75. The total run time of the analytical method was set to 70 min in the HPLC system. The samples were analyzed in triplicate.

### 4.3. RNA Extraction and Library Construction

High-quality transcriptomic RNA was extracted from the leaves using Plant Total RNA Mini Kit with DNase Geneaid^®^. The quality and quantity of the isolated DNA and RNA were checked by agarose gel electrophoresis, NanoDrop 2500 (Thermo Fisher Scientific, USA), and Agilent 2100. The total RNAs of the seven samples were used to construct the RNA-Seq libraries. We used a P5 adaptor sequence P5-AATGATACGGCGACCACCGAGA (5′-3′), P5′-TTACTATGCCGCTGGTGGCTCT (3′-5′), and P7 adaptor sequence CGTATGCCGTCTTCTGCTTG-P7′ (5′-3′), GCATACGGCAGAAGACGAAC-P7 (3′-5′).

### 4.4. Transcriptome Sequencing, Assembly, and Quality Assessment

The transcriptome was sequenced by Novogene Technology using the Illumina HiSeq 2500 platform. After sequencing, the raw reads underwent quality control. Quality control for transcriptomic reads consists of error-rate distribution along with reads and GC content distribution. Raw transcriptomic sequencing data were preprocessed using Filter Tools from Galaxy Platform to remove reads with adaptor contamination, when uncertain nucleotides constitute more than ten percent of either read (N > 10%) and when low-quality nucleotides (base quality less than 5) constitute more than 50% of the reads. High-quality sequencing data were first assembled into contigs and assembled using Geneious Prime 2021.1.1 Align/Assemble tools. Clean reads were de novo assembled by Trinity 2.6.6 software [50] to get assembly transcriptome.

### 4.5. Transcriptomic Reconstruction and Gene Functional Annotation

Trinity 2.6.6 software [50] was used to complete the transcriptome reconstruction process. We used Corset software version-1.09 by Davidson & Oshlack, California, U.S. state (https://github.com/Oshlack/Corset/wiki accessed on 9 September 2021) [51] to develop unigenes. The comprehensive gene functional annotation used seven databases Nr (NCBI non-redundant protein sequences) (Diamond 0.8.22), Nt (NCBI nucleotide sequences) (NCBI blast 2.9.0), Pfam (Protein family) (hmmscan HMMER 3.1), KOG/COG (Diamond 0.8.22), Swiss-Prot (Diamond 0.8.22), KEGG (Kyoto Encyclopedia of Genes and Genome) (Diamond 0.8.22), and GO (Gene Ontology) (Blast2go b2g4pipe_v2.5).

### 4.6. Transcription Factor Analysis, Reference Allignment, and Gene Expression Analysis

iTAK was used to perform the transcription factor analysis of tea. De novo transcriptome filtered by Corset was used as a reference. To map reads back to the transcriptome and quantify the expression level, we used RSEM 1.2.28 [52]. To calculate the gene expression level, RSEM analyzed the mapping results of Bowtie and then got the read count for each gene of each sample. Furthermore, they were converted into FPKM (Fragments Per Kilobase of transcript sequence per Millions of base pairs sequenced) values to estimate the gene expression levels.

### 4.7. Differentially Expressed Genes, GO Enrichment Analysis, and KEGG Pathway Enrichment Analysis

We used edgeR 3.28.0 padj < 0.005 |log_2_(fold change)| > 1 for the differential expression analysis. Volcano plots were used to infer the overall distribution of differentially expressed genes. The Venn diagram presents the number of genes that are uniquely expressed differentially within each group, with the overlapping regions showing the number of genes that are expressed in two or more groups. A major bioinformatics classification system, Gene Ontology (http://www.geneontology.org/ accessed on 9 September 2021), was used to unify the presentation of gene properties across all species. We used GOSeq 1.32.0 software by Young & Davidson, California, U.S. state with a corrected *p*-value of < 0.05 for significant enrichment. For KEGG enrichment, we used KOBAS v3.0 with a corrected *p*-value of <0.05 Pathway enrichment analysis identified significantly enriched metabolic pathways or signal transduction pathways associated with differentially expressed genes compared with the whole genome background (www.genome.jp) accessed on 9 September 2021.

## 5. Conclusions

Transcriptomic analysis identified a large number of potential key regulatory factors that can control the catechin content of shoot tea leaves. KEGG enrichment analyses revealed that the phenylpropanoid, flavonoid, sesquiterpenoid and triterpenoid, alpha-linolenic acid, and drug metabolism-cytochrome P450 metabolic pathways were highly enriched with DEGs. Significant DEGs mainly associated with phenylpropanoid, flavonoid, drug metabolism-cytochrome P450 pathways, and TF (MYB, bHLH, LOB, and C2H2) appeared to be responsible for the high accumulation of secondary metabolites in ‘Kiara-8’ × ‘Sukoi’, ‘Tambi-2’ × ‘Suka Ati’, and ‘Tambi-2’ × ‘TRI-2025’ as was detected in EGCG and catechin content. The content of EGCG and catechin were higher in ‘Kiara-8’ × ‘Sukoi’, ‘Tambi-2’ × ‘Suka Ati’, and ‘Tambi-2’ × ‘TRI-2025’ than in the other hybrids and clone-cultivars. Catechin heterosis, especially in EGCG content in ‘Tambi-2’ × ‘TRI-2025’ compared with female (‘Tambi-2’) and male parents (‘TRI-2025’), and ‘Tambi-2’ × ‘Suka Ati-40’ compared with female parent (‘Tambi-2’). The accumulation pattern of tea quality-related metabolites varied depending on the accessions. These results will also provide candidate genes for the selection of breeding materials in tea plants based on tea quality-related metabolites.

## Data Availability

The data presented in this study are available as Supplementary Files.

## Supplementary Materials

The following supporting information can be downloaded at: https://www.mdpi.com/article/10.3390/plants11151972/s1, Table S1: Data quality control summary of each sample. Table S2: The number of transcripts and unigenes in different length intervals. Table S3: The length distribution of transcripts and unigenes. Table S4: The ratio of successfully annotated genes by each database. Table S5: Five most-common transcription factors. Figure S1: KEGG pathway of phenylpropanoid biosynthesis. Figure S2: KEGG pathway of flavonoid biosynthesis. Figure S3: KEGG pathway of sesquiterpenoid and triterpenoid biosynthesis. Figure S4: KEGG pathway of alpha-linolenic acid metabolism. Figure S5: KEGG pathway of drug metabolism-cytochrome P450.
