# Supplementary material for: Transcriptional Comparison of New Hybrid Progenies and Clone-Cultivars of Tea (Camellia sinensis L.) Associated to Catechins Content
Source: Plants (Basel). 2022 Jul 29;11(15):1972. doi: 10.3390/plants11151972 (PMC9370121; doi:10.3390/plants11151972)
Supplement: Supplementary file 1 [file plants-11-01972-s001.zip › plants-1807949-supplementary.pdf]

Supplementary Files

Table S1. Data quality control summary of each samples.

Sample	Raw reads	Clean reads	Q20(%)	Q30(%)	GC(%)
TRI-2025	42,493,798	41,215,926	97.69	93.29	45.18
Tambi-2	41,598,003	40,395,215	97.64	93.10	44.67
Tambi-1 × Kiara-8	54,426,725	52,877,513	97.91	93.85	44.15
Tambi-2 × Suka Ati-40	44,156,630	42,836,171	97.95	93.78	43.71
Tambi-2 × Cinyiruan-143	41,635,797	40,607,367	97.80	93.50	44.56
Tambi-2 × TRI-2025	44,560,614	43,495,763	97.84	93.61	44.78
Kiara-8 × Sukoi	44,534,876	43,332,030	97.82	93.54	43.98

Table S2. The number of transcripts and unigenes in different length intervals.

Transcript Length Interval	200 bp-500 bp	500 bp-1kbp	1 kbp-2 kbp	>2kbp	Total
Number of transcripts	93,913	104,950	830,030	51,417	333,310
Number of unigenes	43,880	38,269	24,365	15,016	121,530

Table S3. The length distribution of transcripts and unigenes.

Type	Min Length	Mean Length	Median Length	Max Length	N50	N90	Total Nucleotides
Transcripts	301	1,154	794	15,005	1,649	511	384,480,750
Unigenes	301	1,021	651	15,005	1,477	440	124,031,600

Table S4. The ratio of successfully annotated genes by each database.

Statistical items	Number of unigenes	Percentage (%)
Annotated in NR	59,395	48.87
Annotated in NT	85,115	70.03
Annotated in KO	17,010	13.99
Annotated in SwissProt	35,236	28.99
Annotated in PFAM	36,452	29.99
Annotated in GO	36,449	29.99
Annotated in KOG	8,015	6.59
Annotated in all databases	4,713	3.87
Annotated in at least one database	92,472	76.08
Total unigenes	121,530	100

Table S5. Five most-common transcription factor.

TF ID	Subfamily	Family
Cluster-10268.0-1F	GNAT	GNAT
Cluster-10970.0-2R	LOB	LOB
Cluster-11671.0-0F	bHLH	bHLH
Cluster-11862.2-2F	C2H2	C2H2
Cluster-13928.0-2R	MYB	MYB

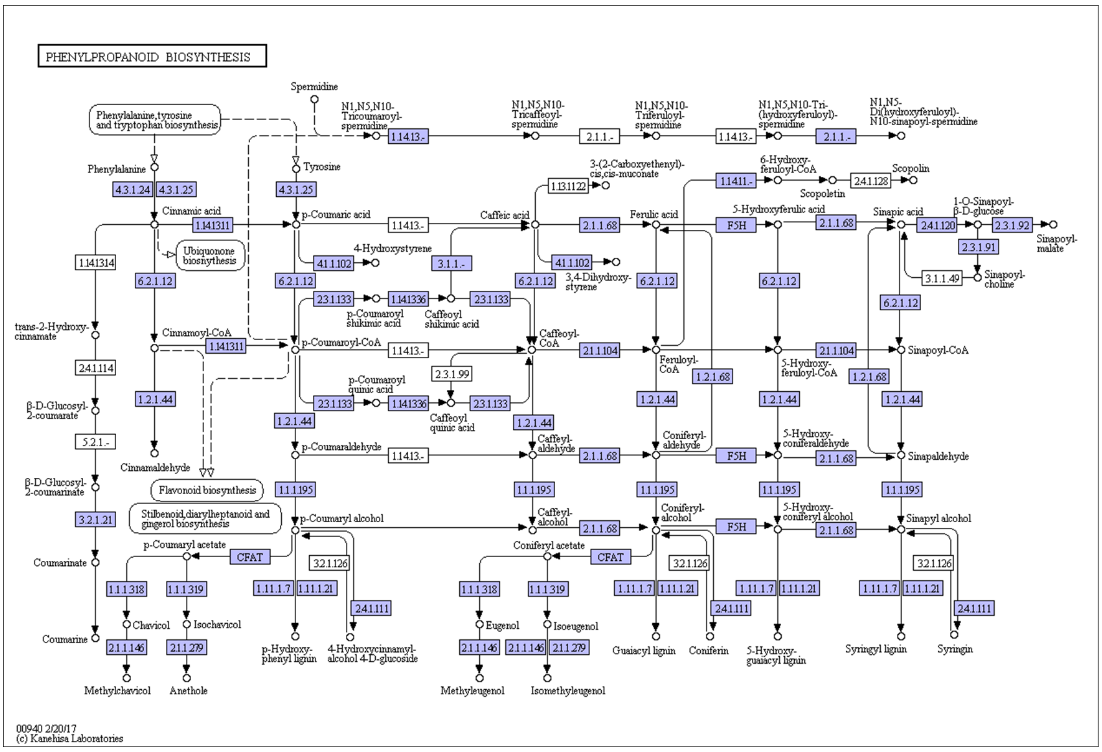

Figure S1. KEGG pathway of phenylpropanoid biosynthesis.

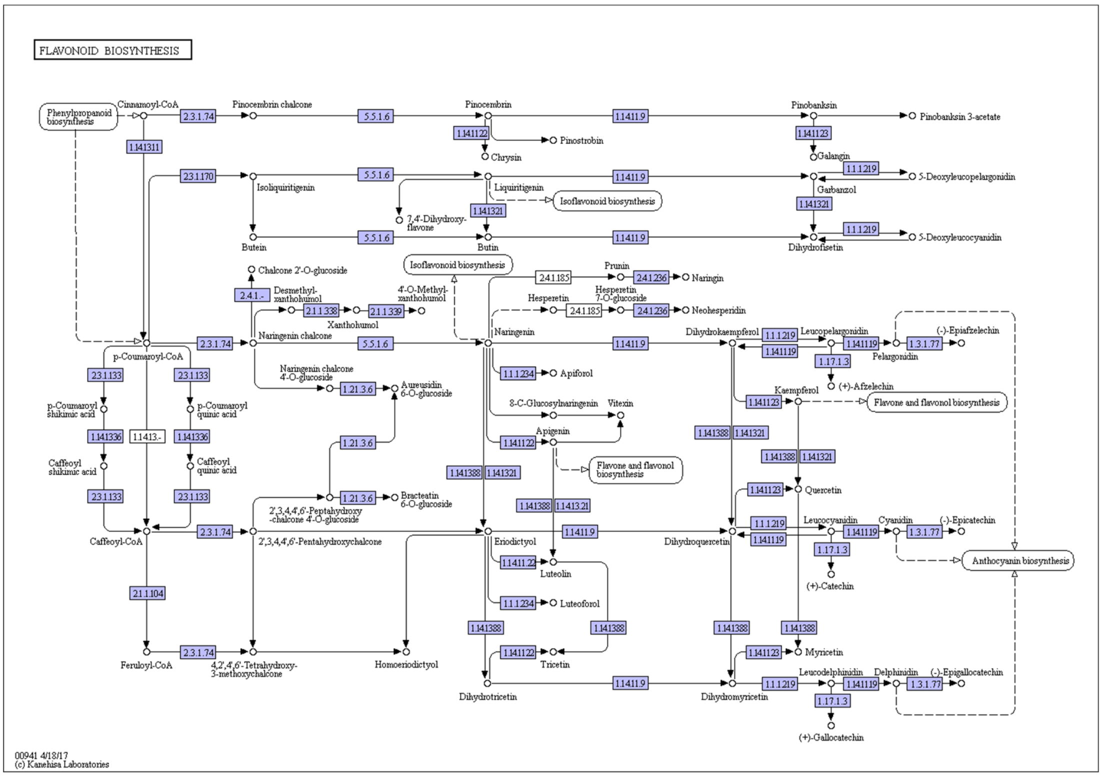

Figure S2. KEGG pathway of flavonoid biosynthesis.

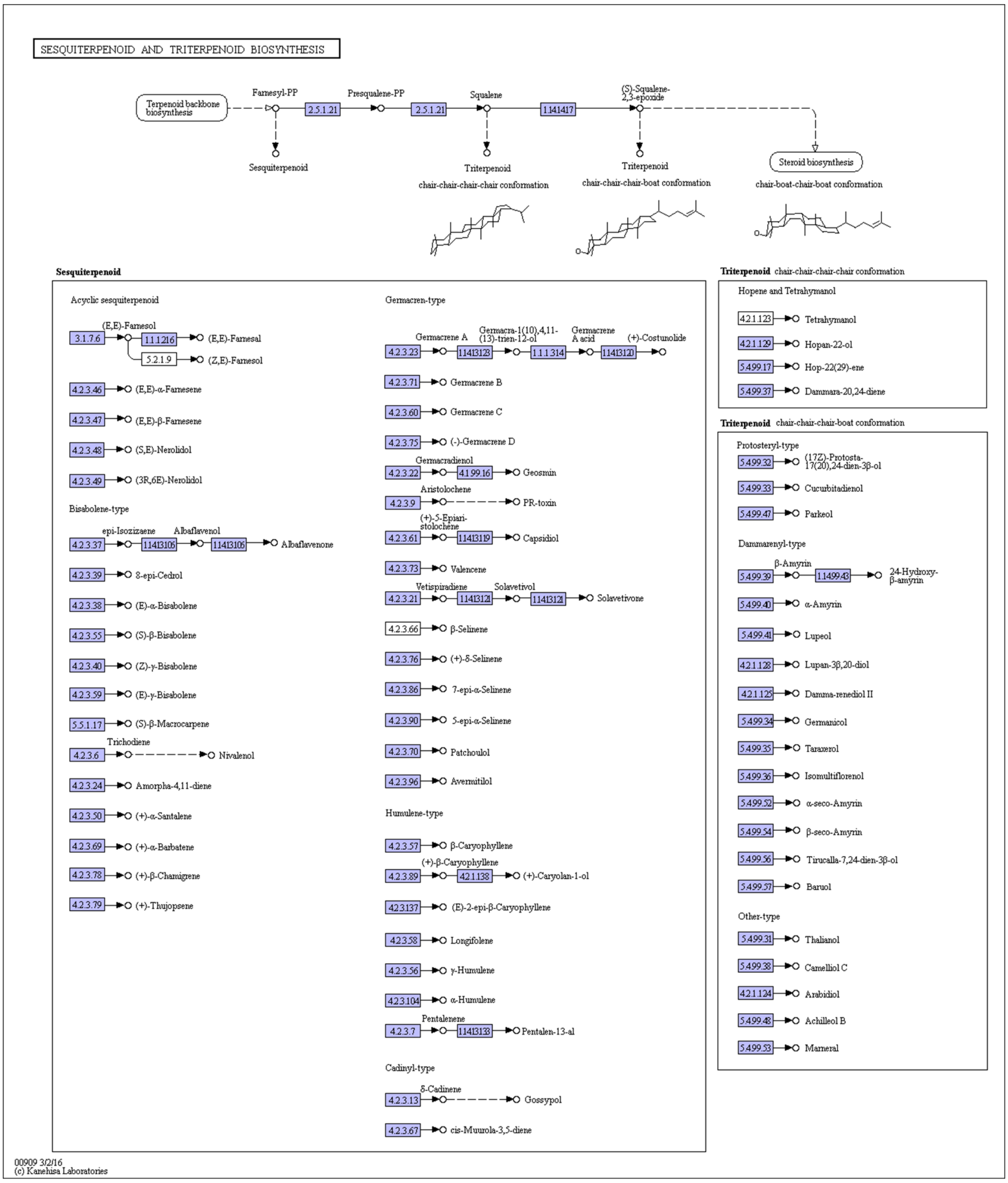

Figure S3. KEGG pathway of sesquiterpenoid and triterpenoid biosynthesis.

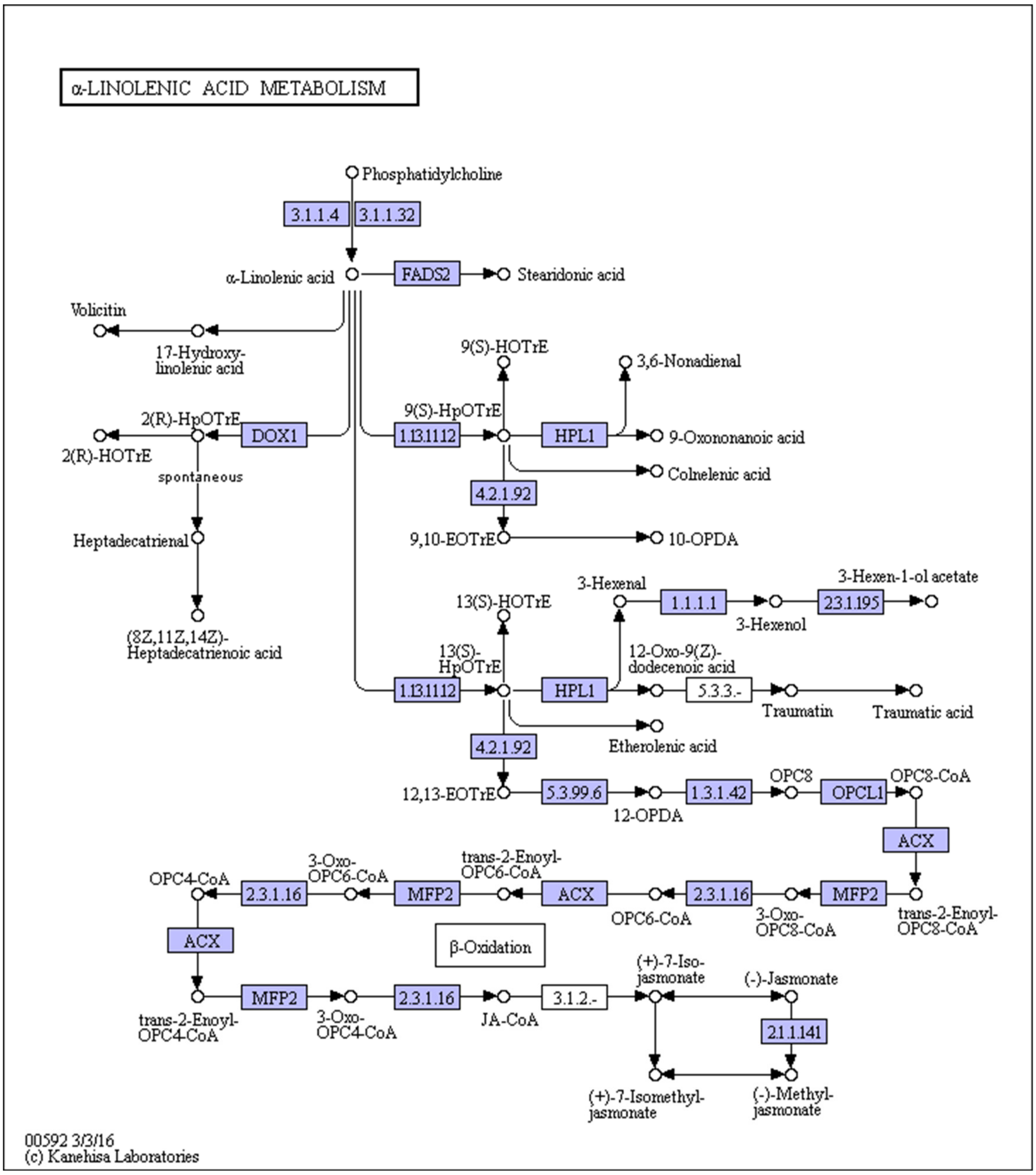

Figure S4. KEGG pathway of alpha-linolenic acid metabolism.

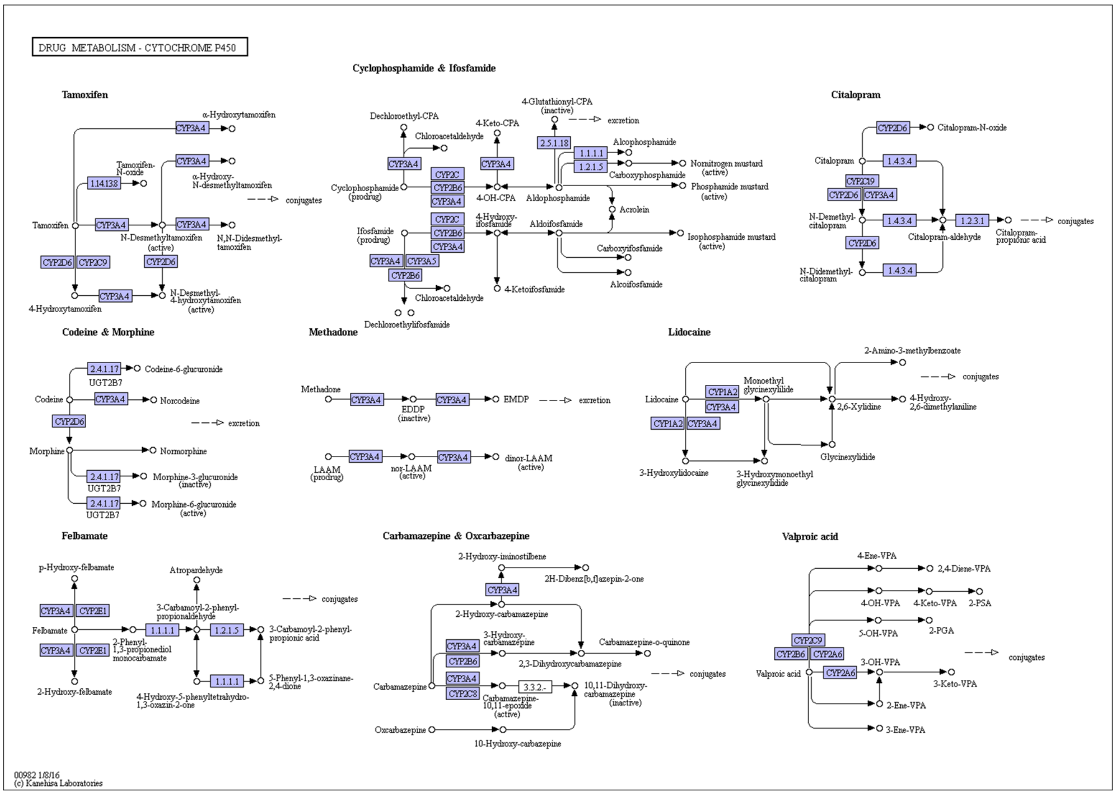

Figure S5. KEGG pathway of drug metabolism-cytochrome P450.
